# Association Between Housing Items and Amenities With Psychological Wellbeing, and Their Possible Impact on All-Cause and Cardiovascular Mortality Risk in Lithuania

**DOI:** 10.3389/ijph.2024.1607356

**Published:** 2024-11-19

**Authors:** Dalia Luksiene, Abdonas Tamosiunas, Giedre Aukstakalniene, Svitlana Boieva, Ricardas Radisauskas, Martin Bobak

**Affiliations:** ^1^ Laboratory of Population Studies, Institute of Cardiology, Medical Academy, Lithuanian University of Health Sciences, Kaunas, Lithuania; ^2^ Public Health Faculty, Medical Academy, Lithuanian University of Health Sciences, Kaunas, Lithuania; ^3^ Laboratory of Molecular Cardiology, Institute of Cardiology, Medical Academy, Lithuanian University of Health Sciences, Kaunas, Lithuania; ^4^ Institute of Epidemiology and Health Care, University College London, London, United Kingdom

**Keywords:** housing items, psychological wellbeing, all-cause mortality, CVD mortality risk, longitudinal study

## Abstract

**Objectives:**

To determine the association of housing items and amenities with psychological wellbeing (PWB) and their relationship with all-cause and cardiovascular disease (CVD) mortality.

**Methods:**

This study was based on the framework of the HAPIEE study. Data from the Lithuanian Mortality Register were used to evaluate CVD and all-cause mortality from baseline survey (2006–2008) till 2023. The logistic regression model and multivariate Cox regression model were applied for data analysis.

**Results:**

The multivariable regression models showed that the material aspects of people’s lives influenced their PWB status: increasing the number of housing items per 1 unit significantly increased the odds ratio (OR) of higher PWB status for males [OR = 1.14 (95% CI 1.11–1.18)] and females [OR = 1.13 (95% CI 1.11–1.17)] and decreased the risk of all-cause and CVD mortality in females [respectively HR = 0.93 (95% CI 0.91–0.96) and HR = 0.91 (95% CI 0.87–0.95)] and in males [respectively HR = 0.92 (95% CI 0.90–0.94) and HR = 0.90 (95% CI 0.87–0.93)].

**Conclusion:**

These data suggest that the household items and amenities influenced PWB and may be used as risk factors in assessing the risk of all-cause and CVD mortality.

## Introduction

Housing items and amenities are important in determining health outcomes. These include basic infrastructure and utilities that make a home safe, comfortable, and conducive to good health and wellbeing [1, 2]. The association between housing items ownership and psychological wellbeing (PWB) can be explained through several psychological, social, and economic mechanisms: housing items ownership affects PWB in various ways, including socioeconomic security, sense of control and autonomy, comfort and convenience, social status, identity expression, and emotional attachment [3, 4]. The ownership of household items like appliances and electronics may reflect financial stability, which can reduce stress and anxiety related to basic and luxury needs. For instance, owning certain household items and amenities (such as smart appliances, luxury electronics, cars, agricultural land, or garden) may signify success or social standing. This can lead to higher self-esteem and greater social recognition, both of which are important contributors to PWB. Together, these factors contribute to an overall improvement in life satisfaction, less stress, and improved PWB and health [1, 5, 6]. Some results of research studies suggest that a higher level of PWB affects long-term survival [7, 8], especially its protective role from cardiovascular (CVD) mortality [9, 10].

Lithuania, like many other European countries, is experiencing demographic changes, including an aging population. Despite earlier diagnosis and treatment of CVD, Lithuania has for many years had the highest mortality rate from CVD among all causes of death. In 2022, in Lithuania according to the data of the Center for Health Information of the Institute of Hygiene (Lithuania), more than half of all deaths, i.e., 52.5%, were due to diseases of the circulatory system [11]. Despite many studies demonstrating traditional risk factors for morbidity and mortality from chronic diseases such as CVD [12], there is an increasing focus on other determinants of health and PWB. For this reason, the aim of this study was to determine the association between housing items and amenities with PWB, and their possible impact on all-cause and CVD mortality risk according to sex.

## Methods

### Study Samples

We used the Lithuanian data of the international project Health, Alcohol and Psychosocial Factors in Eastern Europe (HAPIEE) database as the data source. The HAPIEE study is a prospective cohort study in four countries [13]. In Lithuania, the baseline survey was carried out (during 2006–2008) in Kaunas city males and females aged 45–72 years. The study sample of 10,980 individuals, stratified by sex and age group, was randomly selected from the Lithuanian National population register. In the baseline survey, 7,115 individuals participated (the response rate was 64.8%). All participants were followed up from the baseline survey for all-cause and CVD mortality events until 31st December 2022. We excluded 643 respondents who lacked information on study variables from statistical analysis. Exclusion criteria: respondents for whom the nurses could not take a blood sample due to technical reasons, respondents who refused to give blood for tests, and respondents who did not fill in the questionnaires correctly. Excluded persons in most cases did not differ from the rest of the cohort according to other analysed variables. Finally, a total of 6,472 participants (2,909 males and 3,563 females) were available for statistical analysis. The study was approved by the Kaunas Regional Biomedical Research Ethics Committee, Lithuania (11 January 2005; No. 05/09) and the Ethics Committee at University College London, United Kingdom. All study participants provided written informed consent.

### Sociodemographic, Lifestyle, and PWB Variables

The variables were determined at the baseline survey using the questionnaire by the HAPIEE study protocol [13]. The standard questionnaire of the study also included questions regarding the participant’s sex, age, education, marital status, self-rated health, smoking habits status, etc.

Education was classified into two levels: 1) secondary and lower and 2) college and higher. Participants were classified by marital status as married, cohabitant, single, divorced, and widowed. Self-rated health was assessed by the question: “How would you rate your health in the last 12 months?”. The responses were classified into three categories: poor, good, and very good health. The smoking habits of the participants were assessed according to their current smoking status and classified: as current smokers (regularly smoking at least one cigarette per day), former smokers, and never smokers.

PWB was evaluated by a Control Autonomy Self-realization and Pleasure (CASP-12) questionnaire [14] at the baseline survey 2006–2008. It is composed of 12 statements. Participants indicate how often (often, sometimes, not often, never) each statement applies to them. The total score ranges from 12 to 48, where a higher score represents a higher PWB. The internal consistency of the CASP-12 scale was good (Cronbach’s alpha = 0.74). Participants were labelled as having a higher PWB if their CASP-12 score was higher or equal to the median: ≥40 in males and ≥38 in females.

### Housing Items and Amenities Assessment

The standard HAPIEE study questionnaire included questions regarding the respondent’s ownership of housing items and amenities [13]. In this study 20 housing items and amenities for data analysis were used. A total score for housing items ownership was obtained by summing the items owned by the household. To evaluate the role of housing items and amenities on mortality and PWB, we grouped the housing items and amenities into three groups: “basic needs” (central heating, hot water, central sewage, central water supply, microwave, washing machine, refrigerator, phone or mobile phone); “socially oriented needs” (colour TV, video player, internet at home, personal computer, car), and “luxury” (freezer, dishwasher, satellite or cable TV, DVD player or recorder, video camera/portable video camera with video player, second flat or house, agricultural land or garden) [5]. The housing items ownership and amenities have been measured only once, at the baseline survey 2006–2008.

### Other Covariates

#### Metabolic Syndrome and Its Components

Metabolic syndrome and its components were defined following the Third Report of the National Cholesterol Education Program Adult Treatment Panel III (NCEPATP III) definition [15]. Elevated triglycerides were defined as triglyceride level ≥1.7 mmol/L. Reduced high-density lipoprotein (HDL) cholesterol was defined as <1.0 mmol/L in males and <1.3 mmol/L in females. Hyperglycaemia was defined as a fasting glucose level ≥6.1 mmol/L. Central obesity was defined according to waist circumference (males >102 cm, females >88 cm). Elevated arterial blood pressure was defined as systolic blood pressure >130 mmHg and diastolic blood pressure >85 mmHg. Any three or more components from five were defined as metabolic syndrome.

Waist circumference was measured at the level of the umbilicus using a skin ruler during calm breathing. Blood pressure was measured three times at 2-minute intervals between measurements using an Omron M5-I digital blood pressure monitor. Blood specimens were drawn by trained nurses on a fasting basis. Biochemical analyses for evaluation of lipid concentrations (high-density lipoprotein (HDL) cholesterol and triglycerides) in serum were performed, using a conventional enzymatic method. Serum samples were analysed in the WHO Regional Lipid Reference Centre, Institute of Clinical and Experimental Medicine, Prague (Czech Republic). The glucose concentration in capillary blood was determined by an individual glucometer “Glucotrend” [16].

### Cardiovascular Diseases

CVD included coronary heart disease (CHD) and/or stroke which were determined at the baseline survey. CHD was determined through the following procedures - first, documented history of myocardial infarction (MI) and (or) ischemic changes on electrocardiogram (ECG) coded by the Minnesota codes (MC) 1–1 or 1–2 [17]; second, angina pectoris on effort was defined by the G. Rose questionnaire [without MI and (or) MC 1–1 or 1–2; 3] [18]; third, ECG findings by MC 1–3, 4–1, 4–2, 4–3, 5–1, 5–2, 5–3, 6–1, 6–2, 7–1, 8–3 [without MI and (or) MC 1–1, 1–2 and without angina pectoris]. To assess the history of previous strokes of the participants, a question from the standard questionnaire was asked: “Has a doctor ever told you that you have had a stroke?”. The survey data of previous stroke cases were linked with hospital records.

### Follow-Up for Mortality Outcome

We used data from the Official Lithuanian Mortality Register based on death certificates with follow-up from the start of the baseline survey (2006) until 31 December 2022. Cause of death was categorized using the International Classification of Diseases, 10th Edition (ICD-10). All causes of death were evaluated using ICD-10 codes A00-Z99. CVD-specific mortality was categorized using codes I00-I99. The median follow-up for the endpoint period was 14.5 years in males and 15.3 years in females.

### Statistical Analysis

All statistical analysis was performed using IBM SPSS Statistics (Version 29.0) (IBM Corp. Released 2020. IBM SPSS Statistics for Windows, Version 29.0. Armonk, NY, United States). All continuous variables included in the analysis were tested for normality by skewness and kurtosis values. We performed an analysis of study data separately for males and females. All descriptive characteristics (proportions, means, SD, and medians) were calculated in sex groups. Differences between groups were detected by independent sample t-test for continuous variables that were normally distributed. For variables that did not accept the assumptions of normality, a nonparametric Mann-Whitney test was performed to evaluate the differences between the sex groups. A chi-squared test and z-test were used to assess the differences in categorical variables. Values of *p* < 0.05 were considered statistically significant.

We fit the logistic regression models to estimate the odds ratios (OR) and 95% confidence interval (CI) for three categories of housing items and amenities (basic needs, socially oriented needs, and luxury) with the probability of higher PWB status in males and females. Models included all three housing items and amenities categories each divided into three groups (the group with the lowest number of housing items ownership was a reference group) and were adjusted for sex, age, education, self-rated health, and marital status. Odds of higher PBW status were also assessed using the logistic regression when the total number of housing items and amenities in the models changed per 1 unit.

Hazard ratios (HR) and 95% CI were estimated by multivariate Cox proportional hazards regression for CVD and all-cause mortality in males and females. The risk of all-cause and CVD mortality was calculated for each category of housing items and amenities in one model and adjusted for age, education status, smoking habits status, metabolic syndrome status, and CVD at baseline survey (for CVD mortality only). The risk of CVD and all-cause mortality was also assessed using the Cox regression when the total number of housing items and amenities in the models changed per 1 unit.

## Results

A total of 6,472 participants aged 45–72 years were enrolled in the study, including 2,909 males (44.9%) and 3,563 females (55.1%).

Table 1 shows the baseline characteristics differentiated by sex. The mean age in the male and female groups did not differ at the baseline survey, and the prevalence of some of the biological and lifestyle risk factors in those groups was significantly different. In the male group, only the elevated arterial blood pressure and regular smoking were higher compared to the women group. However, the prevalence of metabolic syndrome and its components such as increased waist circumference and low level of HDL cholesterol, also poor self-rated health were higher and the mean PWB was lower in the female group compared to the male. The prevalence of CVD at the baseline survey in male and female groups did not differ, however, the proportion of death from all causes and death from CVD was about 1.8 times higher (*p* < 0.001) in males compared to females [respectively 17.4% (95% CI 16.0–18.8) and 9.5% (95% CI 8.5–10.5)] during the follow-up period.

**TABLE 1:** Baseline characteristics of men and women at the baseline survey of the Health, Alcohol and Psychosocial Factors in Eastern Europe study, Kaunas, Lithuania, (2006–2008).

Variables	Males	Females	*p*
	N = 2,909	N = 3,563	
Age, years, mean ± SD	60.5 (7.6)	60.4 (7.6)	0.164
Education, %			
Secondary and lower	48.1	40.3	<0.001
College and higher	51.9	59.7	<0.001
Marital status, %			
Married	84.0	56.0	<0.001
Cohabitant	1.4	0.8	<0.001
Single	1.9	5.7	<0.001
Divorced	7.4	15.8	<0.001
Widowed	5.3	21.7	<0.001
Self-rated health, %			
Poor	10.8	17.3	<0.001
Good	56.2	61.6	<0.001
Very good	33.0	21.1	<0.001
Psychological wellbeing, mean ± SD	39.2 (5.5)	37.6 (6.2)	<0.001
Psychological wellbeing groups, %			
Higher	47.1	44.7	0.06
Lower	52.9	55.3	0.06
Regular smoking, %	30.1	9.9	<0.001
Metabolic syndrome, %	28.1	38.0	<0.001
Metabolic syndrome components:			
Elevated arterial blood pressure (≥130/85 mm/Hg), %	85.3	74.2	<0.001
Increased waist circumference, %			
Men ≥102 cm, women ≥88 cm	28.9	53.0	<0.001
HDL cholesterol			
Men <1.0 mmol/L, women <1.3 mmol/L, %	12.0	25.3	<0.001
Triglycerides ≥1.7 mmol/L, %	27.5	27.3	0.909
Fasting glucose ≥6.1 mmol/L, %	32.0	32.5	0.699
Housing items and amenities^a^ (total), mean ± SD	13.2 (3.1)	12.2 (3.1)	<0.001
Housing items and amenities groups:			
Basic needs, number of items, %			
0–4	3.0	3.1	0.408
5–6	10.6	15.3	<0.001
7–8	86.4	81.6	<0.001
Socially oriented needs, number of items, %			
0–1	14.1	29.9	<0.001
2–3	40.0	36.5	<0.001
4–5	45.9	33.6	<0.001
Luxury, number of items, %			
0–1	22.8	32.2	<0.001
2–3	47.8	47.1	0.287
4–7	29.4	20.7	<0.001
Prevalence of CVD at baseline survey, %	20.4	22.3	0.069
Follow-up, median	14.5	15.3	<0.001
Dead from all-causes, %	36.8	19.4	<0.001
Dead from CVD, %	17.4	9.5	<0.001

CVD, cardiovascular diseases; HDL, high-density lipoprotein; SD, standard deviation.

^a^ In this study **20 housing items and amenities** for data analysis were used. We grouped the housing items and amenities into three groups: “**basic needs**” (8 items and amenities: central heating, hot water, central sewage, central water supply, microwave, washing machine, refrigerator, phone or mobile phone); “**socially oriented needs**” (5 items: colour TV, video player, internet at home, personal computer, car), and “**luxury**” (7 items: freezer, dishwasher, satellite or cable TV, DVD player or recorder, video camera/portable video camera with video player, second flat or house, agricultural land or garden).

The mean number of ownership housing items was higher in the male group compared to the female. Also, the prevalence of the increased number of items from three categories of housing items and amenities [“basic needs” (7–8), “socially oriented needs” (2–3 and 4–5), and “luxury” (4–7)] was higher in the male group compared to the female. It was important to determine the association of those housing items with PWB status according to sex and to assess whether the number of housing items could increase the prevalence of higher PWB status.

The material aspects of people’s lives influenced their PWB status, even after being adjusted by age, education, self-esteem, and marital status. Thus increasing the number of housing items per 1 unit increased the odds of higher PWB status for males OR = 1.14 (95% CI 1.11–1.18; *p* < 0.001) and females OR = 1.13 (95% CI 1.11–1.17; *p* < 0.001).

Table 2 shows the possibilities of having higher PWB status by three categories of housing items and amenities (“basic needs,” “socially oriented needs,” and “luxury”) according to sex. Presented data were adjusted for age, education, self-rated health, and marital status.

**TABLE 2:** The odds of higher psychological Wellbeing status by identified categories of housing items and amenities according to sex (Health, Alcohol, and Psychosocial Factors in Eastern Europe study, Kaunas, Lithuania, 2006–2008).

Housing items and amenities	Males			Females			All		
**Groups, the number of items**	OR*	95% CI	p	OR*	95% CI	p	OR**	95% CI	p
Basic needs									
0–4	1			1			1		
5–6	1.05	0.63–1.76	0.850	1.85	1.18–2.90	0.008	1.44	1.02–2.02	0.036
7–8	1.08	0.67–1.75	0.741	2.01	1.32–3.08	0.001	1.53	1.12–2.10	0.008
Socially oriented needs									
0–1	1			1			1		
2–3	1.47	1.15–1.90	0.003	1.32	1.09–1.60	0.004	1.38	1.19–1.61	<0.001
4–5	1.62	1.22–2.15	0.001	1.43	1.14–1.79	0.002	1.49	1.25–1.78	<0.001
Luxury									
0–1	1			1			1		
2–3	1.59	1.29–1.96	<0.001	1.40	1.18–1.66	<0.001	1.48	1.30–1.69	<0.001
4–7	2.30	1.79–2.94	<0.001	1.94	1.53–2.45	<0.001	2.09	1.77–2.48	<0.001

OR* (odds ratios) adjusted for age, education, self-rated health, and marital status.

OR** (odds ratios) adjusted for sex, age, education, self-rated health, and marital status.

CI, confidence interval.

Housing items and amenities groups: “**basic needs**” (8 items and amenities: central heating, hot water, central sewage, central water supply, microwave, washing machine, refrigerator, phone or mobile phone); “**socially oriented needs**” (5 items: colour TV, video player, internet at home, personal computer, car), and “**luxury**” (7 items: freezer, dishwasher, satellite or cable TV, DVD player or recorder, video camera/portable video camera with video player, second flat or house, agricultural land or garden).

There was a significant effect of ownership of basic needs items for increasing odds of higher PWB status only in the female group. However, the increasing number of ownerships of socially oriented needs items and luxury items significantly increased the odds of higher PWB status in male and female groups. In all responder group, (additionally adjusted by sex) the probability of having a higher PBW increased statistically significantly with increasing numbers of housing items and amenities in the basic needs, socially oriented, and luxury groups. Thus, improvements in ownership of housing items contributed to enhanced PWB, and this, in turn, may have positive implications for reducing the all-cause and CVD mortality risk.

Table 3 presents the multivariate association of ownership of housing items with all-cause and CVD mortality in males and females during the follow–up for the endpoints period, after adjustment for age, education status, smoking habits status, metabolic syndrome status, PWB status, and CVD at baseline survey (for CVD mortality only).

**TABLE 3:** Risk of all-cause and cardiovascular mortality by housing items and amenities and psychological Wellbeing groups according to sex (Health, Alcohol and Psychosocial Factors in Eastern Europe study, Kaunas, Lithuania, 2006–2008).

Housing amenities and items, the number of items	Males				Women			
	All-cause	Mortality	CVD	Mortality	All-cause	Mortality	CVD	Mortality
	HR*	95% CI	HR**	95% CI	HR*	95% CI	HR**	95% CI
Basic needs								
0–4	1		1		1		1	
5–6	0.99	0.69–1.40	0.83	0.52–1.31	0.81	0.55–1.18	0.74	0.45–1.21
7–8	0.82	0.60–1.13	0.63	0.42–0.96	0.76	0.53–1.08	0.65	0.41–1.04
Socially oriented needs								
0–1	1		1		1		1	
2–3	0.86	0.72–1.02	0.78	0.61–0.98	0.77	0.64–0.92	0.78	0.60–1.01
4–5	0.67	0.55–0.83	0.56	0.42–0.76	0.84	0.65–1.08	0.73	0.48–1.09
Luxury								
0–1	1		1		1		1	
2–3	0.89	0.76–1.03	0.88	0.70–1.09	0.94	0.79–1.11	1.01	0.79–1.29
4–7	0.74	0.60–0.91	0.71	0.52–0.96	0.66	0.48–0.91	0.57	0.33–0.96
PWB groups								
Lower	1		1		1		1	
Higher	0.79	0.70–0.90	0.86	0.73–1.07	0.84	0.71–0.98	0.78	0.62–0.98

CI, confidence interval; CVD, cardiovascular diseases.

HR* (hazard ratios) adjusted for age, education status, smoking habits, and metabolic syndrome status. Also, models include PWB, groups and all housing items and amenities.

HR** adjusted for all the variables included in HR* for all-cause mortality plus cardiovascular disease (CVD) at baseline survey.

PWB - wellbeing.

Housing items and amenities groups: “**basic needs**” (8 items and amenities: central heating, hot water, central sewage, central water supply, microwave, washing machine, refrigerator, phone or mobile phone); “**socially oriented needs**” (5 items: colour TV, video player, internet at home, personal computer, car), and “**luxury**” (7 items: freezer, dishwasher, satellite or cable TV, DVD player or recorder, video camera/portable video camera with video player, second flat or house, agricultural land or garden).

In the male group the increased number of items from the socially oriented needs group (4–5), and from the luxury group (4–7) compared to items (0–1 in each group) significantly decreased the risk of all-cause mortality (on average by respectively 33% and 26%) and the mortality risk from CVD (in average by respectively 44% and 29%). Also, in the male group of basic needs (7–8) the risk of mortality from CVD was on average 37% significantly lower compared to males with basic items from 0 to 4. In the female group, the luxury items (4–7) compared to items (0–1) significantly decreased the risk of all-cause mortality on average by 34% and the mortality risk from CVD on average by 43%.

Similar significant results were obtained when analysing the influence of the ownership of the number of housing items on all cause-mortality and mortality from CVD risk (data adjusted by age, education status, smoking habits status, metabolic syndrome status, PWB status, and CVD at baseline survey). In the male group increasing the number of housing items per 1 unit decreased the risk of all-cause mortality on average by 8% [HR = 0.92 (95% CI 0.90–0.94; *p* < 0.001)] and the mortality risk from CVD on average by 10% [HR = 0.90 (95% CI 0.87–0.93; *p* < 0.001)]. In the female group increasing the number of housing items per 1 unit decreased the risk of all-cause mortality on average by 7% [HR = 0.93 (95% CI 0.91–0.96; *p* < 0.001)] and the mortality risk from CVD on average by 9% [HR = 0.91 (95% CI 0.87–0.95; *p* < 0.001)].

## Discussion

It’s important to recognize the multifaceted nature of these relationships, as PWB is influenced by various factors, including individual characteristics, social support, and community dynamics. Addressing ownership of housing-related factors that positively impact PWB can be part of a comprehensive approach to promoting overall health and potentially reducing mortality risks associated with CVD and other health conditions.

In this study, we evaluated the relationship between ownership of housing items and amenities and the PWB of the subjects, as well as the significance of both mentioned variables on the risk of total mortality and mortality from CVD. The significance of the increasing number of housing items and amenities for various health indicators has certainly been studied many times, but in the analysis of research data, they were usually not used as independent variables, as we analysed, but they were included together with other signs in various indices, for example, socioeconomic status or HOUSES Index [19, 20]. Also, the important is that housing items and amenities are not more often evaluated as independent health indicators, but more often the housing is analysed as a component of other risk factors such as socioeconomic determinants of health [21]. Thus, the novelty of this study is that we used in the regression models the ownership of housing items and amenities variables as an independent risk factor and evaluated their relationship with the subjects’ PWB, as well as the significance of both mentioned variables for the risk of all-cause mortality and mortality from CVD.

The results from our study indicated that increasing the number of housing items and amenities per 1 unit significantly increased the odds of higher PWB status for males on average by 14% and females on average by 13%, despite that data were adjusted by age, education, self-rated health, and marital status. It’s important, that when housing items and amenities were categorized into three groups (basic needs, socially oriented needs, and luxury) the number of items from those groups was associated with the increased odds of higher PWB status in male (with the exception of “Basic needs“ group) and in female groups. The increasing amount of the number of basic needs items (5–8 compared to 0–4) increased the odds of higher PWB status only in the female group. However, an increased number of items of socially oriented and luxury needs were associated with increased odds of higher PWB status in both male and female groups. In this way, we can say that it is more important for higher PWB of males not to have basic needs that improve the quality of household, but more expensive ones as luxuries that improve the quality of life. It’s crucial to note that these generalizations are broad and may not apply to everyone. Preferences are influenced by various factors such as age, cultural background, personal experiences, and others.

The results from a cross-sectional survey in Pakistan indicated that the number of housing amenities and post-marital status were linked to reduced distress among females exclusively; in contrast, financial problems were identified as major predictors of distress for males [22]. This observation was explained by sex roles in Pakistan, where males are expected to be the primary breadwinners while females are tasked with managing relationships and negotiating their self-identity within intricate family and kinship networks [22]. However, given the cultural disparities and the focus on sex equality in our context, we cannot directly apply or generalize from this example. Unfortunately, we could not find scientific publications reporting results from recent studies analysing the association between housing items and amenities with PWB according to sex and their relationship with all-cause and CVD mortality in the countries closer to Lithuania. For this reason, we presented the results from the study performed in Pakistan. Nonetheless, we hypothesize that different interests among the sexes may contribute to the varying importance of items within the basic needs categories: in men’s group basic needs were not associated with odds of higher PWB in contrast with the women’s group. However, socially oriented and luxury needs remain equally vital for both sexes.

Research on the direct relationship between specific housing items and all-cause mortality risk is complex and often influenced by various factors such as individual health, lifestyle, socioeconomic status, and environmental conditions. Most scientific studies analysed the associations between socioeconomic status, health behaviours of study participants, and all-cause mortality risk [23, 24]. However, certain aspects of housing can indirectly affect overall health and wellbeing, potentially contributing to mortality risk. Findings from Central and Eastern Europe and the former Soviet Union countries (HAPIEE study participants pooled samples) show the inverse graded association between household amenities and mortality: persons who were in the bottom tertile of this variable had the highest mortality, as compared with the persons in the top tertile [23]. The results from the EPIPorto Cohort Study show, that living in substandard or social housing was associated with worse health as measured by mortality [25]. This association remained even after occupation and education were considered, meaning that individuals of the same sex, age, education, and occupation had different mortality risks. Also, was indicated that the link between mortality and poor housing was even stronger than risk factors such as lower education, arterial hypertension, obesity, physical inactivity, heavy alcohol consumption, or heavy physical work [25]. The results of the study performed in Belgium between 1991 and 2020, demonstrated that mortality was associated with housing quality: persons living in normal housing had a lower mortality rate compared to those with poorer housing and greater housing deprivation [26]. However, there is not much direct research or consensus on a specific list of housing items and amenities that have a direct impact on all-cause mortality, we set out in this study to assess how housing items and amenities, as independent risk factors, are associated with the risk of all-cause mortality. The significant results were obtained when we analysed the influence of the ownership of the number of housing items on all-cause mortality. Despite, that Cox proportional hazards regression analysis included many confounders such as age, education status, smoking habits status, metabolic syndrome status, PWB status, and follow-up years, the results show that increasing the number of housing items per 1 unit significantly decreased the risk of all-cause mortality by about 7%–8% in females and males. Once more, the increased number of items from the luxury group significantly decreased all-cause mortality risk in male and female groups, but the increased number of socially oriented needs decreased the all-cause mortality risk only in the male group.

There is also a lack of direct research or consensus on housing items and amenities that have a direct impact on CVD mortality. A systematic review done by Parekh et al. analysed only housing instability (homelessness, affordability, insecurity) and food insecurity such as determinants of cardiovascular outcomes [27]. The recent results from the longitudinal analysis of CVD mortality in US counties show, that housing and food insecurity have caused chronic stress and led to a host of chronic diseases (such as hypertension, diabetes, depression, and anxiety) that have increased the risk of CVD-related diseases and mortality [28]. Despite, that 2021 ESC Guidelines on Cardiovascular Disease Prevention in Clinical Practice emphasized that cardiovascular health is a complex outcome influenced by a combination of genetic, lifestyle, and environmental factors [12], we set the goal to evaluate how housing items and amenities as independent risk factors are associated with the risk of mortality from CVD. Significant results were obtained when we analysed the influence of the ownership of the number of housing items on mortality from CVD risk. Despite, that Cox proportional hazards regression analysis included many confounders, including CVD at baseline survey the results show that increasing the number of housing items per 1 unit significantly decreased the risk from CVD by about 9%–10% in female and male groups. Also, the increased number of items from the luxury group significantly decreased mortality from CVD risk. Results of the study performed in Israel also show that the household amenities variable was highly predictive of socioeconomic inequalities in overall and cardiovascular disease mortality in males and females [29, 30].

While traditional socioeconomic factors remain crucial to understanding social health inequalities in different population groups, our research emphasizes the not deeply analysed the significant role of housing items and amenities. By treating them as independent variables, we offer a more nuanced understanding of how housing items and amenities are associated with higher PWB and risk of non-communicable diseases. Our research could be evaluated as groundbreaking in exploring how housing items and amenities not only complement but also interact with more traditional risk factors of non-communicable diseases. So, understanding the complex interplay between material factors such as ownership of housing items and psychological mechanisms is crucial for developing holistic strategies to promote health and wellbeing. Researchers and policymakers are increasingly recognizing the need for multidisciplinary approaches to address the diverse factors influencing health outcomes.

### Strengths and Limitations

The main strength of this study is its prospective design and large sample size. Other strengths include data collection using standardized and validated research methods, a long follow-up period, and many potential confounders included in the statistical analysis (7 variables in fully adjusted data from multivariate regression models).

This study also has some limitations. First, despite adjusting for multiple confounders, we did not include a family history of CVD in the statistical models, which may have influenced the study results. Second, this study relied on some self-reported outcomes (PWB, health status, and smoking habits), which may be affected by recall bias and may have overestimated or underestimated the findings. Third, the housing items ownership and PWB have been measured only once, at the baseline survey 2006–2008, for this reason we could not evaluated the changes of these variables during life of responders. Fourth, we do not know exactly what diseases or comorbidities our subjects had during the follow-up period, what new chronic disease risk factors and harmful lifestyles emerged during that period, and for how long.

### Conclusion

These data suggested that an increasing number of owned housing items and amenities, as a material aspect of people’s lives, influenced their PWB and could be applied as - risk factors for the evaluation of all-cause and CVD mortality risk. For those reasons addressing housing-related factors that positively impact PWB can be part of a comprehensive approach to promoting overall health and potentially reducing mortality risks associated with CVD and other health conditions.
